# Digital Phenotyping: A Game Changer for the Broiler Industry

**DOI:** 10.3390/ani13162585

**Published:** 2023-08-10

**Authors:** Suresh Neethirajan

**Affiliations:** Department of Animal Science and Aquaculture, Faculty of Computer Science, Dalhousie University, Halifax, NS B3H 4R2, Canada; sneethir@gmail.com

**Keywords:** animal health monitoring, broiler genomics, broiler industry, digital phenotyping, digital twins, multi-modal digital phenotyping platform, production resilience, poultry production

## Abstract

**Simple Summary:**

This study explores the potential of digital phenotyping, a technological innovation, to transform the broiler industry. It aims to address industry challenges such as productivity, animal welfare, and environmental impacts. The research focuses on the use of ‘digital twins’ in broiler genomics and the development of a digital phenotyping platform for health monitoring. Despite potential technical, economic, and ethical challenges, the study concludes that digital phenotyping could revolutionize the industry, striking a balance between productivity and sustainability. The insights from this study could guide future research and development, leading to a more robust and sustainable broiler industry.

**Abstract:**

In response to escalating global demand for poultry, the industry grapples with an array of intricate challenges, from enhancing productivity to improving animal welfare and attenuating environmental impacts. This comprehensive review explores the transformative potential of digital phenotyping, an emergent technological innovation at the cusp of dramatically reshaping broiler production. The central aim of this study is to critically examine digital phenotyping as a pivotal solution to these multidimensional industry conundrums. Our investigation spotlights the profound implications of ‘digital twins’ in the burgeoning field of broiler genomics, where the production of exact digital counterparts of physical entities accelerates genomics research and its practical applications. Further, this review probes into the ongoing advancements in the research and development of a context-sensitive, multimodal digital phenotyping platform, custom-built to monitor broiler health. This paper critically evaluates this platform’s potential in revolutionizing health monitoring, fortifying the resilience of broiler production, and fostering a harmonious balance between productivity and sustainability. Subsequently, the paper provides a rigorous assessment of the unique challenges that may surface during the integration of digital phenotyping within the industry. These span from technical and economic impediments to ethical deliberations, thus offering a comprehensive perspective. The paper concludes by highlighting the game-changing potential of digital phenotyping in the broiler industry and identifying potential future directions for the field, underlining the significance of continued research and development in unlocking digital phenotyping’s full potential. In doing so, it charts a course towards a more robust, sustainable, and productive broiler industry. The insights garnered from this study hold substantial value for a broad spectrum of stakeholders in the broiler industry, setting the stage for an imminent technological evolution in poultry production.

## 1. Introduction

Projected to burgeon to a staggering $429.11 billion by 2028 with a compound annual growth rate (CAGR) of approximately 5.45% from 2022 [1], the global chicken market’s escalated growth has imposed an acute burden on the broiler industry. This sector faces the daunting task of sustainably and efficiently satisfying this surging demand. At this significant juncture, the industry finds itself uniquely positioned to harness revolutionary technologies, such as digital phenotyping [2,3], which emerges as a key contender in transforming this landscape.

As the broiler industry strives to enhance production efficiency, it simultaneously grapples with the imperative of minimizing environmental impact [4]. Concurrently, a heightened global awareness of animal welfare compels the industry to reevaluate and adjust traditional practices. Within this complex web of challenges, digital phenotyping offers a potent transformative solution.

### 1.1. Digital Phenotyping: The Forefront of Innovation

Digital phenotyping is defined as the “moment-by-moment quantification of the individual-level human (or animal) phenotype in situ using data from personal digital devices” [5]. This concept is closely related to the field of Precision Livestock Farming (PLF), a term coined by Daniel Berckmans. PLF involves the use of advanced technologies to enhance the efficiency and sustainability of livestock production by providing precise, real-time monitoring and management of individual animals [6]. In essence, digital phenotyping can be seen as a key component of Precision Livestock Farming, enabling the collection and analysis of detailed, individual-level data to optimize livestock health, welfare, and productivity.

Digital phenotyping, a pioneering intersection of technology and biology, heralds promising avenues for the broiler industry [7]. By exploiting the capabilities of digital phenotyping, the poultry sector can access profound insights into bird health, growth, and behavior, promoting optimized production, enhanced animal welfare, and environmentally sustainable practices. Thus, it is crucial to probe the nuances of digital phenotyping and critically assess its implications for the broiler industry.

In essence, digital phenotyping involves harnessing digital technologies to monitor phenotypic traits—the observable physical and behavioral characteristics of an organism, molded by its genetic composition and environment. The application of sophisticated tools such as Artificial Intelligence (AI), Machine Learning (ML), and Internet of Things (IoT) devices in digital phenotyping introduces a dynamic mechanism for real-time data collection and analysis [8].

### 1.2. The Digital Phenotyping Matrix: An In-depth Analysis

IoT devices, strategically deployed within poultry houses, gather an extensive array of data related to the birds’ environment, including temperature, humidity, and air quality [9]. Simultaneously, tools powered by Artificial Intelligence (AI), including its subsets Machine Learning (ML) and Deep Learning (DL), meticulously analyze the birds’ activity patterns. These AI-enabled tools can detect deviations in behavior, monitor physical growth, and provide critical insights into the birds’ health and well-being.

Yet, this potent amalgamation of technology and poultry farming, despite appearing as an ideal panacea for the industry’s pressing challenges, presents its own complexities upon closer and more critical inspection. While digital phenotyping bears the potential to drastically alter the landscape of the broiler industry, it is not a panacea for all industry issues. The integration of these advanced technologies requires substantial investment, specialized skills, and a robust infrastructure.

Moreover, the implementation of digital phenotyping technologies necessitates robust data management systems and formidable data security measures [10]. Given the increasing prevalence of cyber-attacks in the modern digital world, securing the voluminous quantities of sensitive data gathered by these technologies emerges as a vital concern.

### 1.3. The Digital Phenotyping Verdict: An Equilibrated Perspective

Digital phenotyping offers a compelling path for the broiler industry to optimize production, elevate animal welfare, and alleviate environmental impact. Yet, its success critically hinges on the cautious and responsible application of these technologies, buttressed by robust data management and security measures. As the broiler industry stands on the precipice of this technological revolution, it must navigate the path ahead with prudent foresight, rigorous evaluation, and an unwavering commitment to sustainable practices.

Indeed, the real transformative force will not merely be the technologies themselves, but how the industry leverages them to propel sustainable and efficient growth [11]. While the complete narrative of digital phenotyping’s success in the broiler industry is yet to be written, the initial chapters indeed promise an intriguing saga of progress, challenges, and transformative potential.

This critical review aims to address the following objectives:

- Illuminating the Concept of Digital Twins in Broiler Genomics: This objective entails an exploration of the concept of digital twins in broiler genomics and an assessment of their potential to revolutionize the industry. The goal is to ascertain how digital twins can augment our understanding of the genetic constitution of broiler chickens and improve breeding strategies for desirable traits.
- Investigating the Development of a Multi-modal, Context-aware Digital Phenotyping Platform for Broiler Health: This objective involves a comprehensive evaluation of research and development efforts focused on creating a multi-modal digital phenotyping platform custom-built for broiler health monitoring. The aim is to assess the efficacy of various digital phenotyping technologies, such as IoT devices, AI algorithms, and image analysis techniques, in capturing and analyzing phenotypic traits pertinent to broiler health.
- Advancing Broiler Health: A Multi-modal Digital Phenotyping Platform for Comprehensive and Efficient Health Monitoring: Investigate how a multi-modal digital phenotyping platform can contribute to comprehensive and efficient health monitoring in the broiler industry. This objective aims to highlight the potential benefits of digital phenotyping in early disease detection, behavioral analysis, growth monitoring, and overall welfare assessment of broiler chickens.
- Enhancing Resilience in Broiler Production through Digital Phenotyping: This objective focuses on analyzing how digital phenotyping can bolster the resilience of broiler production systems. The aim is to comprehend how digital phenotyping can provide valuable insights into environmental conditions, optimize feed management, and improve overall production efficiency amidst environmental and economic challenges.
- Addressing the Challenges of Digital Phenotyping in the Broiler Industry: This objective involves a critical evaluation of the challenges and limitations encountered by the broiler industry in adopting and implementing digital phenotyping technologies. The goal is to identify and address hurdles such as high investment costs, data management complexities, cybersecurity risks, and the necessity for specialized skills and infrastructure.

By fulfilling these objectives, this review paper aims to furnish critical insights into the role of digital phenotyping in the broiler industry and its potential to transform broiler genomics, health monitoring, and overall production systems. It also aims to illuminate the challenges faced by the industry and propose recommendations for the responsible and effective implementation of digital phenotyping technologies.

## 2. Global Demand for Poultry and the Challenges Faced by the Broiler Industry

Positioned at a crucial juncture with escalating global demand for poultry products, the broiler industry is facing an array of challenges requiring astute consideration and strategic solutions. The burgeoning demand exerts tremendous pressure on the industry, necessitating an increase in production efficiency while preserving environmental sustainability. Simultaneously, the sector grapples with a rising consciousness towards animal welfare and a complex regulatory landscape, further complicated by prevalent disease risks and balancing cost-effective production [12,13]. As we explore the potential remedies to these challenges, digital twins in broiler genomics emerges as a promising solution.

### 2.1. Balancing Efficiency and Environmental Sustainability

To accommodate the surging demand for poultry products, the industry must innovate to boost production without exacerbating environmental concerns such as deforestation, water scarcity, and greenhouse gas emissions [14,15]. Neglecting these issues could lead to harmful environmental impacts and jeopardize the industry’s long-term viability.

### 2.2. Prioritizing Animal Welfare

With consumers becoming increasingly aware of broiler chickens’ rearing conditions and intensive farming practices’ effects, the industry is experiencing a shift towards ethically sourced and humanely raised poultry products [16]. Failing to address these concerns may lead to reputational damage and the loss of consumer trust.

### 2.3. Navigating Regulatory Landscape

The broiler industry, like many other sectors, is subject to a myriad of regulations and standards that vary across different regions and countries. This regulatory landscape becomes particularly challenging to navigate for small-scale farmers who may lack the necessary resources and expertise to ensure compliance. The advent of digital phenotyping in the broiler industry introduces an additional layer of complexity to this landscape.

Digital phenotyping, while offering transformative potential for the industry, also brings with it a host of new considerations around data usage and protection. The data collected through digital phenotyping are often sensitive, encompassing detailed information about individual animals and farming practices. The responsible usage and protection of these data is paramount, not only to maintain the trust of farmers and other stakeholders but also to ensure the ethical application of this technology.

However, as digital phenotyping is a relatively new field, there is a conspicuous lack of comprehensive regulations and guidelines specifically tailored to its unique challenges. Existing data protection regulations may not fully address the nuances of data usage in digital phenotyping, leaving potential gaps in protection.

This regulatory vacuum poses a significant challenge for the industry. Without clear guidelines, there is a risk of inconsistent data practices, which could undermine the reliability of digital phenotyping and potentially infringe on data protection rights. Furthermore, the absence of regulations could stifle innovation, as uncertainty around permissible data practices may deter investment and research in this field.

Therefore, there is an urgent need for the development of comprehensive regulations and guidelines that can guide the responsible usage and protection of data in digital phenotyping. These regulations should balance the need for data protection with the potential benefits of digital phenotyping, providing clear and practical guidance for all stakeholders in the broiler industry.

Navigating the regulatory landscape is a critical task for the broiler industry as it embraces digital phenotyping. The development of comprehensive regulations and guidelines will be key to ensuring the responsible and ethical use of this promising technology.

### 2.4. Managing Disease Risks

Disease outbreaks, such as avian influenza, pose significant risks to both bird health and public health, leading to economic losses and public health concerns. Therefore, investments in robust biosecurity measures, disease surveillance, and vaccination programs are crucial, and advances in digital phenotyping and data analytics play a pivotal role in early disease detection and proactive risk management.

### 2.5. Cost-Effective Production

Reconciling the need for affordable poultry products with escalating production costs, the industry must optimize production processes, minimize waste, and investigate cost-effective alternatives without compromising quality or safety.

## 3. The Advent of Digital Twins in Broiler Genomics: A Revolutionary Concept with Potential to Reshape Industry

In the wake of genomics and digital technology advancements, breeding programs within the poultry industry have witnessed significant improvement over the years. Amidst this progress, the concept of a digital twin—a virtual representation of a physical entity or system, facilitating testing, prediction, and optimization in a secure environment—emerges as a potent game-changer. This technology, serving as a linchpin of industry advancement, bears the potential to revolutionize broiler breeding.

The primary objective of a digital twin resides in the creation of a dynamic virtual duplicate of a physical or biological entity or process [17,18]. In the realm of broiler breeding, this translates to the generation of a model simulating genetic selection, accounting for specific traits and delivering predictive insights. Essentially, this simulation model becomes a prognostic tool for various breeding strategies’ outcomes. In the subsequent section, we delve meticulously into the intricate, step-wise process of developing a digital twin model for broiler genomics, traits, and breeding strategies.

### 3.1. Step 1: Data Aggregation—The Cornerstone of Digital Twin Development

The cornerstone of digital twin development lies in the comprehensive collection of data pertaining to broiler genetics and performance traits. Genetic data constitutes information such as DNA sequences, whereas performance traits encompass factors such as growth rate, body size, meat quality, and disease resistance [19].

DNA Sequencing: Contemporary genotyping and sequencing technologies provide a myriad of methods for genetic data acquisition. From simple SNP (Single Nucleotide Polymorphism) arrays to exhaustive whole-genome sequencing, each technique possesses its own set of advantages and trade-offs concerning cost, accuracy, and depth of information. The decision to adopt a specific method hinges on the unique objectives and available resources of the breeding program.

Phenotypic Data: Concurrent to genetic data, it is imperative to compile comprehensive phenotypic data concerning the birds. This includes observable characteristics such as body weight, feed conversion ratio, and disease resistance, emerging from the birds’ genetics interacting with their environment.

Environmental Data: Acquiring information about the environmental conditions in which the birds are raised is indispensable. This data can incorporate factors such as diet, temperature, humidity, and other elements influencing gene expression and overall performance.

### 3.2. Step 2: Twin Model Development—The Genesis of a Predictive Mechanism

With the collected data, the next phase involves creating a digital twin model accurately reflecting the genetic architecture and prospective evolution of broilers. This step necessitates deploying mathematical and computational tools to simulate the genetic and phenotypic diversity of the broiler population, in conjunction with selection and breeding processes. This process entails several sub-steps:

Genetic Model: The genetic model should mirror the established and inferred associations among different genetic markers and traits. This requires the application of statistical techniques such as quantitative trait loci (QTL) mapping and genome-wide association studies (GWAS) to identify the genetic variants linked to each trait.

Breeding Model: The breeding model should emulate the selection process, considering factors such as the mating strategy (e.g., random mating, assortative mating), the number of offspring per pair, and the selection intensity and criteria.

Integration of Genetics and Environment: Ultimately, the model should amalgamate genetic and environmental factors to predict phenotypic outcomes. This necessitates comprehending how distinct genes and environmental conditions interact to influence traits, attainable via techniques such as genotype-by-environment interaction (GxE) analysis.

### 3.3. Step 3: Simulation and Predictive Analysis—Navigating the Landscape of Probabilities

Upon developing the digital twin, it can be employed to simulate an array of scenarios and predict various breeding strategies’ outcomes. These prognostications inform decisions about prioritizing certain traits, selecting birds for breeding, and managing the genetic diversity of the population.

Running Simulations: The digital twin can be used to simulate multiple broiler generations under diverse breeding scenarios. For instance, one could simulate the impact of intense selection for growth rate versus balancing growth rate with other traits such as disease resistance.

Predictive Analysis: By juxtaposing the outcomes of different simulations, the breeding strategies that are most likely to accomplish your objectives can be identified. This process demands sophisticated data analysis techniques, ranging from traditional statistical methods to advanced machine learning algorithms.

Sensitivity Analysis: Conducting sensitivity analyses to understand how modifications in different parameters (e.g., selection intensity, mating strategy) impact outcomes is also pivotal. This can aid in identifying the key factors driving genetic improvement and the potential trade-offs between different objectives.

### 3.4. Step 4: Optimization—Achieving Balance in a World of Trade-Offs

Based on predictive analysis results, it is feasible to optimize the breeding strategies [20]. The aim is to pinpoint strategies that maximize desired outcomes (e.g., improved growth rate, better meat quality) while minimizing undesired ones (e.g., increased susceptibility to diseases, reduced genetic diversity).

Multi-objective Optimization: Given that breeding programs generally have multiple objectives [21], it is crucial to utilize multi-objective optimization techniques. These techniques can balance competing objectives and help identify the “Pareto frontier”—the set of strategies offering the best trade-offs among different goals.

Genetic Algorithms: Genetic algorithms, which mimic the process of natural selection, are a formidable tool for optimization [22]. They can search a vast space of possible strategies and find the ones that perform optimally according to the chosen criteria.

### 3.5. Step 5: Real-World Application and Feedback—Refining the Mirror of Reality

The final step involves implementing the optimal breeding strategies in the real world and then refining the digital twin based on the results. This step is critical to ensuring that the digital twin retains accuracy and relevance as conditions change and new data becomes available.

Following these steps, it is feasible to create a robust and versatile digital twin for broiler breeding, thereby enhancing the efficiency and effectiveness of genetic selection processes. The ability to predict and optimize outcomes could significantly expedite genetic improvement in broilers, resulting in healthier, more productive birds, and a more sustainable poultry industry [23].

This approach offers numerous benefits. It enables more precise and effective selection, potentially boosting productivity and profitability. It can help circumvent the risk of unexpected negative consequences from certain breeding choices. It can accelerate genetic improvements by predicting the impact of different breeding strategies and reduces the need for physical trial and error, conserving valuable time and resources.

However, this approach also presents challenges, including the requirement for voluminous, high-quality data, the complexity of creating accurate digital models, and the difficulty of integrating genetics with other relevant factors such as nutrition and environmental conditions [24,25]. Nevertheless, with advances in data science and genomics, the potential of digital twins in broiler breeding is becoming increasingly achievable.

In the pursuit of advancing the broiler industry through digital innovation, our study introduces a novel approach to leveraging digital phenotyping for comprehensive monitoring and analysis. As delineated in Table 1, this approach encompasses a wide array of methodologies and technologies, all aimed at enhancing the understanding of broiler health and behavior. This integrative perspective not only offers a detailed insight into the current state of broiler health but also paves the way for future advancements in the field.

## 4. Illuminating the Potential of Virtual Reality in Broiler Breeding: A Multifaceted Exploration towards Enhanced Industry Practices

With the evolution of agritech—the fusion of agriculture and technology—agricultural and livestock farming practices are undergoing significant transformation. Among the innovative technological developments, Virtual Reality (VR)—typically acclaimed for its revolutionary applications in gaming and entertainment— has started showcasing its transformative potential across various scientific fields [26,27]. As the domain of animal husbandry experiences a gradual shift towards precision farming, trailblazing technologies such as VR have the potential to redefine our path towards enhanced productivity and sustainability.

This research trajectory elucidates the exploration of VR application in broiler breeding, a crucial segment of the global poultry market. While breeding technologies have progressed significantly, the sector continues to grapple with challenges such as the selection of superior parent stock, maintenance of genetic diversity, and disease control. The focal point of this research trajectory is the innovative utilization of VR [28,29] for a holistic analysis of genomic data in broiler breeding, which could facilitate informed breeding decisions.

With global demand for poultry products experiencing an exponential surge, the broiler industry finds itself burdened with an unprecedented mandate. Simultaneously grappling with the escalating demand and the imperative to lessen environmental impact, the industry stands at a complex juncture. With growing consumer concern for animal welfare demanding a paradigm shift in traditional practices, VR’s potential application in broiler breeding emerges as a beacon of transformation in this intricate landscape.

Digital phenotyping, an innovative convergence of technology and biology, is gathering momentum as a potential disruptor for the broiler industry. With this pioneering technology, the poultry sector could gain invaluable insights into bird health, growth, and behavior, fostering optimized production, improved animal welfare, and sustainable practices.

The prime objective of is to explore the multi-faceted potential of VR in broiler breeding. It aims to address a series of questions:

- Does VR provide an effective tool to understand broiler behavior and interaction within a controlled virtual environment?
- Can complex genomic data and molecular networks be made comprehensible and interactive through VR?
- Can the use of VR enhance decision making in the selection of parent animals for the next generation?

To accomplish these objectives, I propose a multi-dimensional approach incorporating various methodologies leveraging VR’s transformative power.

The first methodology involves implementing VR in visual learning experiments. Drawing inspiration from the work of Geng et al. with bees [30,31], the proposal recommends the utilization of VR to design experiments for understanding broiler behavior. By creating a controlled virtual environment, researchers can observe and record broiler behavior, gaining invaluable insights into their phenotypic traits and adaptability.

The second methodology concentrates on the interactive exploration of genome-scale molecular networks using VR. Building upon the foundational work of Pirch et al. [32], I propose the development of a VR platform to visualize and interact with complex molecular networks integral to broiler breeding. This platform will facilitate a deeper comprehension of intricate genetic factors influencing broiler breeding.

Data visualization and analysis form the cornerstone of the third methodology. Following the pioneering work of Stein et al. [33] and Legetth et al. [34], I propose the utilization of VR for an in-depth examination of complex genomic data. By leveraging VR platforms for modeling RNA velocity and analyzing single-cell data, researchers can derive insightful conclusions for broiler breeding, which can guide more informed decision making.

The fourth methodology involves the virtual simulation of gametes. Inspired by the study of Bijma et al. [35], I propose the application of VR to simulate broiler gametes, thus increasing the probability of breeding top-ranking genotypes based on gametic variability. This groundbreaking approach has the potential to significantly enhance breeding outcomes, producing more resilient and disease-resistant broilers.

Further, development of detailed subgene-level maps using VR as a critical part of this research would open up novel perspectives. Harnessing the power of VR, researchers can create comprehensive and interactive subgene-level maps of broiler genomes, enabling a quicker identification of crucial genes responsible for productivity and health. This will provide valuable insights that can drive more targeted and efficient breeding strategies.

The expected outcomes of this study are manifold. The research aims to generate insights into broiler behavior and their interaction in a VR environment, shedding light on their adaptability and responses to various stimuli. The development of a VR platform for visualizing and understanding complex genomic data and molecular networks will deepen the understanding of genetic factors influencing broiler breeding. The virtual simulation of gametes has the potential to revolutionize breeding outcomes, enabling the selection of top-ranking genotypes based on gametic variability. Lastly, the subgene-level map of broiler genomes will expedite the identification of genes crucial for productivity and health, leading to more targeted and efficient breeding strategies.

However, the integration of VR in broiler breeding, while promising, is not without its challenges. Key issues include ensuring the availability of high-quality and comprehensive data, creating accurate virtual models, and integrating genetics with other relevant factors such as nutrition and environmental conditions. Nonetheless, with advancements in data science and genomics, realizing the potential of VR in broiler breeding is increasingly attainable.

Leveraging VR technology, researchers can gain valuable insights into broiler behavior, analyze complex genomic data, simulate gametes, and develop comprehensive subgene-level maps. These advancements hold the potential to revolutionize breeding outcomes, enhance animal welfare, and contribute to the sustainability of the broiler industry. However, careful considerations and further research are required to address the challenges and ensure the responsible and effective implementation of VR in broiler breeding practices.

As the broiler industry continues to face the ever-increasing global demand for poultry products, embracing innovative technologies such as VR can pave the way for a more efficient, sustainable, and productive future. The potential of VR in broiler breeding is vast, and this research proposal aims to uncover its transformative power for the benefit of the industry and its stakeholders.

## 5. Exploring the Development of a Context-Aware, Multimodal Digital Phenotyping Platform for Enhanced Broiler Health Management

Health disorders among broilers can cast significant adverse effects on production, leading to reduced efficiency, heightened mortality rates, and compromised product quality. Traditional diagnostic methods for broiler health disorders often require labor-intensive, manual processes and lack consistency, hindering timely and effective detection and management of health issues [36]. Consequently, the development of advanced, objective assessment technologies capable of providing real-time monitoring and efficient diagnosis of broiler health conditions is imperative.

### 5.1. Objective

This research trajectory seeks to critically investigate efforts directed towards the research and development of a multimodal digital phenotyping platform tailored for broiler health monitoring. The aim is to assess the efficacy of various digital phenotyping technologies, including IoT devices, AI algorithms, and image analysis techniques, in capturing and analyzing phenotypic traits pertinent to broiler health.

### 5.2. Approach

**Figure 1:**
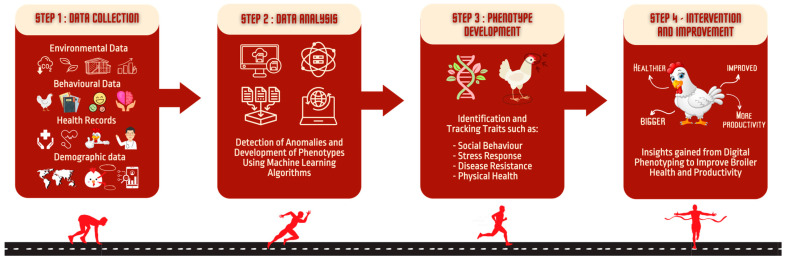
Digital Phenotyping Workflow in the Broiler Industry.

The proposed research project seeks to tackle these challenges via the development of a multimodal, context-aware digital phenotyping platform for broiler health. The research focuses on three pivotal objectives:

- Developing and Validating Context-aware Multimodal Digital Biomarkers: This aspect involves utilizing wearable sensors such as RFID tags and accelerometers, alongside cameras, to collate real-time data relating to broiler behavior and environmental factors. Gathering data on parameters such as movement, vocalization, temperature, and humidity helps establish context-aware biomarkers that can detect latent health issues in broilers across different life stages. Researchers will deploy wearable sensors on broilers to monitor their behavior and vital signs. Additionally, cameras installed within the broiler houses will provide visual information assessing elements such as feather condition, gait, and social interactions. By integrating this multimodal data, researchers can formulate context-aware biomarkers indicative of the health status of the broilers.
- Developing and Validating Multimodal, Multi-domain Digital Phenotypes of Poultry Disorders: The research intends to analyze the gathered data to derive digital phenotypes associated with various health disorders in broilers. The integration of data from diverse modalities, including genetic, phenotypic, and environmental information, allows the creation of comprehensive digital phenotypes. This enables more accurate and predictive assessments of broiler health. The process involves analyzing the data collated from the wearable sensors and cameras utilizing advanced AI algorithms and machine learning techniques. By identifying patterns and correlations within the data, researchers can formulate digital phenotypes that capture the complex interplay between genetic factors, environmental conditions, and health outcomes [37,38,39]. These digital phenotypes will offer a comprehensive understanding of the broiler health status, thereby aiding in early detection and prevention of disorders. Figure 1 shows the process of collecting data from various sources, analyzing these data to detect anomalies and develop phenotypes, and applying these insights to improve broiler health and productivity.
- Development of an Integrated Platform: This objective involves the creation of an integrated software platform that amalgamates algorithms designed to extract biomarkers, latent classes, and digital phenotypes from wearable technologies and cameras. The platform will offer real-time monitoring, analysis, and visualization capabilities, thereby rendering the data accessible and interpretable for broiler health management. Researchers aim to design and implement a software platform that integrates the data from wearable sensors and cameras. The platform will encompass AI algorithms and machine learning models to process and analyze the data in real-time, providing visualizations and actionable insights to broiler farmers, enabling them to make informed decisions regarding broiler health management.

### 5.3. Implications

The successful realization of a multimodal, context-aware digital phenotyping platform for broiler health will carry significant implications for the broiler industry. The platform will facilitate a more objective, comprehensive, and real-time assessment of broiler health conditions, enabling early detection of disorders and prompt intervention. Consequently, it will contribute to improved production efficiency, reduced mortality rates, and enhanced animal welfare. By facilitating data-driven decision making in broiler health management, the platform will empower farmers to optimize their breeding strategies, monitor the influence of environmental factors, and enhance overall broiler performance.

### 5.4. Future Directions

Upon development and validation, the multimodal digital phenotyping platform can be extended for broader applications in poultry breeding. The insights derived from the platform can inform the selection of healthier and more productive broiler lines, leading to improved genetic traits and overall flock performance. Moreover, the technologies and approaches developed through this research can potentially be adapted for use in other livestock sectors, thereby contributing to advancements in precision livestock farming and animal health management.

The research and development of a multimodal, context-aware digital phenotyping platform for broiler health holds tremendous potential for transforming the broiler industry. By leveraging IoT devices, AI algorithms, and image analysis techniques, this platform will enable real-time monitoring, comprehensive analysis, and predictive insights into broiler health conditions. It will empower broiler farmers with the tools and knowledge required to optimize production, enhance animal welfare, and ensure the delivery of high-quality poultry products to meet the growing global demand.

## 6. Establishing a Multi-Modal Digital Phenotyping Framework for Comprehensive, Real-Time Health Monitoring

As the poultry industry moves towards more digital and data-driven practices, the concept of digital phenotyping platforms is growing in relevance and utility. Such platforms harness the power of sensor technology, advanced camera systems, and sophisticated machine learning algorithms to redefine how broiler health management is approached. This exploration delves into the mechanics and merits of these digital phenotyping platforms, shedding light on their transformative potential in augmenting broiler health.

The central objective of this research is to facilitate a more holistic understanding of broiler health, growth, development, and physiological nuances by conceiving an innovative digital phenotyping platform. This platform would exploit the strengths of the latest sensor technologies, high-resolution camera systems, and advanced machine learning algorithms to decode a wide range of broiler phenotypes. This can pave the way to uncover latent health issues and subsequently refine broiler productivity and welfare standards.

### 6.1. Constructing Efficient Computational Pipelines for Phenotype Analysis

An integral aspect of the platform’s development lies in the creation of proficient, high-throughput computational pipelines. These pipelines are meticulously engineered to analyze the data harvested from sensors and cameras, and consequently quantify significant phenotypes in broilers. By applying computational algorithms and statistical models, these pipelines can decode complex data sets, deriving meaningful insights [40,41] to guide informed decision making.

The design of these pipelines is collaborative in nature, relying on shared expertise from research colleagues, data scientists, and research engineers. This cohesive dynamic ensures the consideration of the industry’s priority measurement needs during the development process. This tailored approach ensures that the computational pipelines are well-equipped to confront the unique challenges associated with broiler health monitoring.

### 6.2. Streamlining the Pipeline Creation Process with Modular Design Principles

A parallel objective of the project revolves around streamlining the pipeline creation process. This requires the creation process to be as formulaic and modular as possible, promoting efficiency and standardization. By emphasizing the deployment of existing tools on research cyberinfrastructure, the project accelerates the development and deployment of computational pipelines. This strategy reduces the time and effort required to operationalize new technologies.

By adopting a formulaic and modular approach, the expected outcome ensures the swift and effective creation of new pipelines. This paves the way for broader adoption of digital phenotyping platforms within the broiler industry. By focusing on deploying these existing tools on research cyberinfrastructure, the end result can accelerate the development and deployment of computational pipelines, reducing the time and effort required to implement new technologies.

### 6.3. Leveraging the Potential of Machine Learning Techniques

Machine learning techniques are instrumental in analyzing complex data sets and drawing valuable insights from them. By integrating machine learning algorithms into the computational pipelines, phenotype analysis can be automated and made more accurate [42,43]. Supervised learning algorithms can be trained on labelled data to identify specific phenotypes, such as unusual behavior patterns or disease symptoms. Additionally, unsupervised learning algorithms can be employed to discern hidden patterns and relationships within the data, providing a more comprehensive view of broiler health.

### 6.4. Integration with Research Cyberinfrastructure

To facilitate scalability and ensure that the computational pipelines are readily accessible, integration with research cyberinfrastructure is essential. This involves leveraging cloud computing resources, distributed computing frameworks, and data storage solutions to manage the large volumes of data generated during broiler research. By utilizing these technologies, researchers can process and analyze data in a distributed and parallel manner, significantly reducing the computational time needed for phenotype analysis.

### 6.5. Implications and Future Trajectories

The successful development of a multi-modal digital phenotyping platform for broiler health will have profound implications for the broiler industry. By enabling real-time, comprehensive, and objective assessments of broiler health, early detection and timely interventions for health disorders can be facilitated. This will not only boost productivity but also contribute towards improved animal welfare. The digital phenotyping platform will aid in data-driven decision making, enabling farmers to optimize their management strategies, monitor environmental impacts, and enhance overall broiler performance.

Moving forward, this digital phenotyping platform can be extended for a broader range of applications in poultry breeding. The platform’s insights can guide the selection of healthier and more productive broiler lines, leading to improved genetic traits and overall flock performance. Furthermore, the technologies and methodologies developed through this research can potentially be adapted for other livestock sectors, propelling advances in precision livestock farming and animal health management.

**Figure 2:**
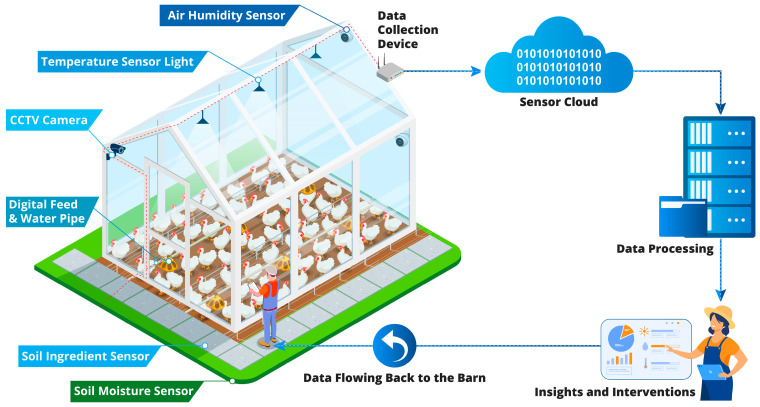
The Digital Phenotyping Ecosystem in the Broiler Industry.

By employing this multi-modal digital phenotyping platform, stakeholders can facilitate the real-time monitoring and comprehensive analysis of broiler health. As a result, the broiler industry can anticipate higher productivity, enhanced animal welfare, and a more sustainable future. The evolution of such data-driven strategies will continue to reshape the landscape of the broiler industry, making the effective use of technology a cornerstone of its success. The strategic integration of sensor technology, high-resolution camera systems, and advanced machine learning algorithms thus forms the crux of our approach. Figure 2 underscores the importance of a scalable, integrated multi-user platform in facilitating this process, highlighting the seamless flow of information from the farm to the server and back, thereby enabling the real-time application of insights for the betterment of the broiler industry.

## 7. Harnessing the Transformative Potential of Digital Phenotyping for Enhanced Resilience in Broiler Production

Amidst the escalating challenges posed by diseases, environmental factors, and climate change, the resilience of broiler production emerges as a critical priority. Broiler resilience encompasses the capacity of birds to withstand and adapt to stressors, maintain robustness, and sustain productivity even in the face of adverse conditions [44,45]. This section presents a comprehensive analysis of how digital phenotyping can significantly bolster the resilience of broiler production. By delving into the potential impacts on efficiency, productivity, animal welfare, and environmental sustainability, we aim to elucidate the transformative power of digital phenotyping in fortifying broiler resilience.

### 7.1. Holistic Understanding of Broiler Resilience

Broiler resilience embodies a multifaceted concept that encompasses robustness, adaptability to diseases, and the ability to withstand environmental and climate variations. Robustness refers to the unwavering ability of broilers to maintain stable performance and optimal health despite fluctuations in their surrounding conditions [46]. Adaptability to diseases entails the capacity to resist or swiftly recover from pathogenic challenges, thereby minimizing the impact on production. Moreover, broilers must demonstrate adaptability to changing environmental circumstances, encompassing temperature variations, humidity levels, air quality fluctuations, and the consequences of climate change.

### 7.2. Unleashing the Potential of Digital Phenotyping for Enhanced Resilience

Digital phenotyping, armed with its remarkable ability to capture and analyze vast volumes of data, presents unparalleled potential for enhancing broiler resilience. By harnessing advanced technologies, including Internet of Things (IoT) devices, artificial intelligence (AI), and machine learning (ML), digital phenotyping empowers real-time monitoring and analysis of phenotypic traits that are pivotal for bolstering resilience.

### 7.3. Revolutionizing Disease Detection and Management

Digital phenotyping platforms have the transformative ability to revolutionize disease detection and management in broiler production. By continuously monitoring crucial indicators such as behavior, activity patterns, and physiological parameters, these platforms can promptly alert farmers to potential health issues at their early stages. Timely detection enables proactive intervention, curbing the spread and impact of diseases and amplifying the resilience of the broiler population.

### 7.4. Empowering Environmental Monitoring and Adaptation

The integration of IoT devices and cutting-edge sensor technologies within digital phenotyping paves the way for comprehensive environmental monitoring. Continuous measurement of temperature, humidity, air quality, and other pertinent environmental factors yields invaluable insights into the profound influence of the environment on broiler health and performance. Armed with these data, farmers gain the power to optimize housing conditions, ventilation systems, and other crucial environmental parameters, thereby augmenting broiler resilience and mitigating the detrimental effects of climate change.

### 7.5. Maximizing Performance Optimization

Digital phenotyping ushers in a new era of precise monitoring of broiler performance, encompassing critical factors such as growth rate, feed efficiency, and carcass quality. By analyzing these data in real-time, farmers can identify potential bottlenecks, inefficiencies, or variations in performance that may compromise resilience. Appropriate adjustments to management practices, nutrition regimens, or breeding strategies can then be implemented to maximize broiler performance and bolster overall resilience.

### 7.6. Fostering Optimal Welfare Assessment

Broiler welfare stands as an integral component of resilience, given that stressed or inadequately managed birds are more susceptible to diseases and environmental challenges. Digital phenotyping empowers a comprehensive approach to welfare assessment by monitoring behavioral patterns, mobility, and social interactions. The early identification of welfare issues facilitates prompt intervention, ensuring optimal welfare conditions and fostering resilience.

### 7.7. Impacts on Efficiency, Productivity, Animal Welfare, and Environmental Sustainability

The application of digital phenotyping within broiler production yields profound implications for efficiency, productivity, animal welfare, and environmental sustainability. By harnessing the inherent power of digital phenotyping, farmers gain the ability to optimize resource utilization, curtail input costs, and minimize waste. Real-time monitoring and proactive intervention lead to improved productivity, enhanced animal welfare, and reduced environmental footprints. Moreover, the data collected through digital phenotyping facilitate evidence-based decision making and cultivate a culture of continuous improvement within the broiler industry.

The transformative potential of digital phenotyping in bolstering the resilience of broiler production is undeniable. By leveraging advanced technologies and data-driven insights, farmers can proactively monitor and manage crucial aspects of broiler resilience, encompassing disease resistance, environmental adaptation, performance optimization, and welfare enhancement. Embracing digital phenotyping as a strategic tool within broiler production paves the way for a more resilient and sustainable future for the industry.

## 8. Specific Challenges That the Broiler Industry Faces in the Field of Phenotyping

The broiler industry faces unique challenges when implementing digital phenotyping, a revolutionary approach to monitoring and optimizing broiler health. Here, I critically examine these challenges, ranging from technical limitations to economic and ethical considerations, and explores potential solutions. By addressing these hurdles, the industry can unlock the full potential of digital phenotyping and strengthen broiler production’s resilience. This comprehensive analysis aims to provide insights into how digital phenotyping can enhance efficiency, productivity, animal welfare, and environmental sustainability in the broiler industry.

### 8.1. Challenges in Digital Phenotyping for Broiler Production

#### 8.1.1. Lack of High-Throughput and Automated Techniques

Traditional phenotyping methods are labor-intensive, time-consuming, and unsuitable for large-scale operations. To overcome this challenge, the industry needs to invest in advanced technologies, such as sensor networks and computer vision, to automate data collection and enable real-time monitoring on a large scale. Implementing high-throughput and automated techniques will streamline the phenotyping process, allowing efficient monitoring of thousands of birds simultaneously.

#### 8.1.2. Inconsistency and Subjectivity in Phenotypic Data

Manual phenotyping is prone to observer bias and variability, resulting in inconsistencies in collected data. To address this challenge, standardized protocols and guidelines should be developed and implemented industry wide. Moreover, integrating computer vision and artificial intelligence (AI) algorithms can automate data collection and minimize human subjectivity, leading to more reliable and consistent phenotypic data. By standardizing data collection and analysis, the industry can ensure accurate comparisons and correlations across different levels.

##### Data Accuracy

In the rapidly evolving field of digital phenotyping, the utilization of data to discern and predict individual responses and well-being is a cornerstone of our research. However, it is crucial to acknowledge that the data harnessed for digital phenotyping can often be characterized by a high degree of noise, potentially obscuring the accurate representation of an individual’s response or state of well-being.

This inherent noise in the data can stem from a multitude of sources, including but not limited to, variability in sensor readings, discrepancies in data collection methods, and the inherent complexity of biological systems. Such noise can introduce a level of uncertainty that may compromise the precision of our findings and predictions, thereby underscoring the pressing need for enhanced accuracy in this domain.

As we delve deeper into the realm of digital phenotyping, it becomes increasingly evident that our quest for accuracy is not merely a pursuit of scientific rigor, but a necessity to ensure the reliability and validity of our findings. The ability to accurately interpret and predict individual responses and well-being through digital phenotyping holds transformative potential for the poultry industry, promising advancements in efficiency, productivity, animal welfare, and environmental sustainability.

Therefore, our focus must be on developing sophisticated data processing and analysis techniques that can effectively filter out the noise and extract meaningful insights from the data. By doing so, we can enhance the accuracy of digital phenotyping, thereby unlocking its full potential to revolutionize the poultry industry.

The pursuit of greater accuracy in the data used for digital phenotyping is not just a scientific imperative but a critical step towards realizing the transformative potential of this technology in the poultry industry.

#### 8.1.3. Limited Ability to Measure Complex Traits

Traditional methods struggle to accurately measure complex phenotypic traits, such as behavior, gait, and social interactions, which are crucial indicators of broiler health and productivity. Developing novel technologies, such as wearable sensors and advanced imaging methods, can enable the measurement of these complex traits in a non-invasive and accurate manner. Additionally, machine learning algorithms can assist in extracting meaningful insights from complex phenotypic data, facilitating a deeper understanding of broiler health and well-being.

#### 8.1.4. Difficulty in Early Detection of Health Issues

Traditional phenotyping methods often detect health problems only after the onset of clinical symptoms, leading to significant impacts on broiler health and productivity. Early detection of diseases or stress conditions through predictive phenotyping can revolutionize broiler health management. Implementing real-time monitoring systems integrated with machine learning algorithms can enable the early detection of health issues by analyzing subtle changes in phenotypic patterns. This proactive approach allows for timely intervention and improved broiler resilience.

#### 8.1.5. Limited Integration of Phenotypic and Genotypic Data

Integrating genotypic data with phenotypic data remains a challenge, yet it is crucial for predicting how genetic changes might affect observable traits in broilers. Developing robust bioinformatics pipelines and data integration frameworks can bridge the gap between genotypic and phenotypic data, facilitating a deeper understanding of the genetic basis of broiler traits. This integration allows for targeted breeding strategies that enhance productivity and resilience in broiler populations.

#### 8.1.6. Lack of Computational Tools and Expertise

The successful implementation of digital phenotyping relies on computational tools, expertise, and data analysis capabilities. However, there is a notable lack of such resources in the broiler industry. To address this challenge, investments should be made in developing user-friendly computational tools and platforms that integrate data analytics, machine learning, and visualization techniques. Additionally, training programs and collaborations between academia and industry can help build the necessary expertise in advanced technologies and data analysis.

### 8.2. Harnessing the Power of Digital Phenotyping for Enhanced Resilience

To overcome these challenges and harness the power of digital phenotyping for enhanced resilience in broiler production, a multi-faceted approach is needed. The industry should focus on:

#### 8.2.1. Research and Development

Invest in research and development efforts to create advanced phenotyping technologies tailored specifically for the broiler industry. This includes the development of wearable sensors, computer vision systems, and AI algorithms optimized for broiler health monitoring.

#### 8.2.2. Collaboration and Knowledge Sharing

Foster collaborations among industry stakeholders, researchers, and technology providers to share expertise, data, and best practices. Such collaborations can accelerate the development and adoption of digital phenotyping solutions, leading to improved broiler health and productivity.

#### 8.2.3. Standardization and Quality Control

Establish industry-wide standards and guidelines for phenotypic data collection, analysis, and interpretation. This ensures consistency and comparability across different farms and research studies.

#### 8.2.4. Data Privacy and Ethics

Address concerns related to data privacy and ethics by implementing secure data management systems and adhering to ethical guidelines for data collection and use. Transparency and responsible data handling practices will build trust among stakeholders and support the sustainable implementation of digital phenotyping.

#### 8.2.5. Education and Training

Promote education and training programs to equip industry professionals with the necessary skills to utilize digital phenotyping technologies effectively. This includes training in data analysis, machine learning, and advanced phenotyping techniques.

By addressing the specific challenges associated with digital phenotyping in the broiler industry, stakeholders can unlock the potential for enhanced resilience in broiler production. Through research, collaboration, standardization, and skill development, the industry can leverage digital phenotyping to optimize broiler health, improve productivity, ensure animal welfare, and promote sustainability.

## 9. Dissecting the Intricacies of Digital Phenotyping: Advantages, Roadblocks, and the Path Forward

It is crucial to delve into the technical intricacies, potential roadblocks, and future prospects of this innovative methodology.

### 9.1. Advantages of Digital Phenotyping in the Broiler Industry

Digital phenotyping offers a plethora of advantages that can significantly impact the broiler industry. Firstly, it allows for real-time and continuous data collection, providing timely insights into broiler health, growth, and behavior. This enables proactive decision making and interventions to optimize productivity and welfare.

Moreover, digital phenotyping enhances the accuracy and reliability of phenotypic measurements. Traditional manual methods are susceptible to observer bias and inconsistency, leading to unreliable data. By automating data collection and analysis, digital phenotyping minimizes human error and ensures standardized and objective measurements.

Another advantage lies in the ability of digital phenotyping to capture complex phenotypic traits. Traditional methods struggle to measure intricate characteristics such as behavior [47], gait, and social interactions, which are crucial indicators of broiler health and productivity. With advanced technologies such as computer vision and AI algorithms, digital phenotyping enables the comprehensive assessment of these complex traits. Table 2 provides a comprehensive breakdown of the digital phenotyping platform components that are instrumental in enhancing broiler health monitoring. By dissecting the intricate elements of the platform, we offer a clear understanding of the insights, benefits, and solutions that digital phenotyping brings to the broiler industry. This detailed exploration underscores the transformative potential of digital phenotyping, emphasizing its role in fostering a more sustainable and efficient approach to broiler health and management.

### 9.2. Roadblocks and Challenges in Implementing Digital Phenotyping

Despite its immense potential, the implementation of digital phenotyping in the broiler industry faces several roadblocks. One significant challenge is the lack of high-throughput and automated techniques in phenotyping. Traditional methods are labor-intensive, time-consuming, and unsuitable for large-scale commercial operations. To fully leverage digital phenotyping, scalable and automated solutions must be developed to handle the high volume of data generated in commercial broiler production. Another obstacle is the inconsistency and subjectivity in phenotypic data. Manual phenotyping methods are prone to observer bias and variability, resulting in inconsistent and unreliable data. Establishing standardized protocols and guidelines for data collection and analysis is crucial to ensure consistency and enable meaningful comparisons across different levels of broiler production.

### 9.3. Robust Data Management and Cybersecurity Measures

As digital phenotyping involves the collection and storage of sensitive data, robust data management and cybersecurity measures are essential. Broiler producers must prioritize data security and privacy to protect against potential breaches and misuse. Implementing encryption, access controls, and regular cybersecurity audits will ensure the integrity and confidentiality of the data.

### 9.4. The Future of Digital Phenotyping in the Broiler Industry

Looking ahead, digital phenotyping holds great potential for transforming the broiler industry. Continued research and development efforts are vital to address the current challenges and unlock the full capabilities of this technology. Collaboration between industry stakeholders, researchers, and technology experts is essential to drive innovation, knowledge sharing, and the development of practical solutions tailored to the specific needs of the broiler industry.

Integrating digital phenotyping with emerging technologies, such as virtual reality, genetic analysis, and precision nutrition, opens up exciting possibilities for further enhancing broiler health, productivity, and sustainability. However, a balanced and insightful perspective is crucial as these advancements unfold. Ethical considerations, data privacy, and animal welfare must remain central in the adoption and application of digital phenotyping.

Digital phenotyping represents a transformative approach to broiler industry monitoring and analysis. Its advantages in real-time data collection, accurate phenotypic measurements, and assessment of complex traits are undeniable. By addressing the roadblocks and challenges through robust data management, cybersecurity measures, and ongoing research, digital phenotyping has the potential to revolutionize the broiler industry, optimizing productivity, animal welfare.

### 9.5. The Imperative for Robust Scientific Validation of Digital Markers

As the broiler industry increasingly adopts digital phenotyping, the reliance on digital markers to assess various aspects of poultry health and welfare becomes more pronounced. These digital markers, derived from a multitude of data sources, serve as the foundation for the insights and predictions generated by digital phenotyping. However, the validity of these markers is contingent upon robust scientific validation, a criterion that some current markers may not fully meet.

The scientific validation of digital markers is a rigorous process that seeks to establish a reliable correlation between the marker and the attribute it is intended to measure. This process often involves extensive empirical testing and statistical analysis, aimed at demonstrating that the marker can accurately and consistently measure the attribute across a variety of conditions.

However, as digital phenotyping is a relatively new field, some of the digital markers currently in use may not have undergone this rigorous validation process. This lack of robust scientific validation can cast doubt on the reliability of these markers in assessing health and welfare, among other attributes. Without reliable markers, the insights and predictions generated by digital phenotyping may be skewed or inaccurate, potentially leading to suboptimal decisions and outcomes.

This issue underscores the need for ongoing research and validation efforts in the field of digital phenotyping. As we continue to develop and refine digital markers, it is crucial that we also invest in rigorous scientific validation to ensure their reliability. This will involve not only empirical testing and statistical analysis but also peer review and replication studies to confirm the validity of our findings.

The robust scientific validation of digital markers is a critical aspect of digital phenotyping that requires our sustained attention and effort. By ensuring the reliability of our markers, we can enhance the accuracy and utility of digital phenotyping, paving the way for its successful application in the broiler industry.

## 10. Summary and Conclusions

This analysis elucidates the transformative potential of digital phenotyping, an innovative paradigm poised to redefine the broiler industry through the fusion of advanced technologies such as sensor technology, computer vision, machine learning, and data analytics. Such a technological amalgamation proffers a ground-breaking framework for the meticulous scrutiny and optimization of broiler health, productivity, and resilience. The integration of digital phenotyping within the broiler industry, however, is not devoid of intricate challenges. These include a deficiency in high-throughput, automated methods; subjectivity and inconsistency in phenotypic data; a restrained capability in quantifying intricate traits; obstacles in early health issue detection; limited integration of phenotypic and genotypic data; and a scarcity of computational tools and adeptness. Notwithstanding, through meticulous research and development, fruitful collaborations, standardization endeavors, and educational and training initiatives, these constraints are surmountable.

The conception of specialized multi-modal digital phenotyping platforms for broiler production could herald significant advancements. These platforms promise to automate the phenotyping process, bolster data accuracy and consistency, enable the measurement of intricate traits, expedite early health issue detection, and amalgamate phenotypic and genotypic data. By confronting these challenges and harnessing the transformative power of digital phenotyping, the broiler industry could potentiate enhanced resilience, heightened efficiency, superior animal welfare, and increased environmental sustainability.

The ramifications of digital phenotyping are substantial, endorsing data-driven decision making in broiler health management. This allows agricultural practitioners to refine their breeding strategies, monitor the repercussions of environmental factors, and bolster overall broiler performance. The integration of digital twins in broiler genomics, the creation of multi-modal and context-aware phenotyping platforms, and the exploration of virtual reality applications, all represent burgeoning avenues for future research and innovation.

Digital phenotyping potentially represents a seismic shift for the broiler industry, offering an integrated and efficient mechanism to surveil broiler health, amplify productivity, enhance animal welfare, and ensure sustainability. By addressing the present challenges and capitalizing on the opportunities proffered by digital phenotyping, the broiler industry can pave the way for a more resilient and prosperous future. A continuous commitment to research, collaboration, and investment in cutting-edge technologies and expertise remains critical to fully unleash the revolutionary potential of digital phenotyping within the broiler industry.

## Figures and Tables

**Table 1:** Leveraging Digital Phenotyping for Comprehensive Monitoring and Analysis in the Broiler Industry.

Digital Phenotyping Applications	Specific Use in Broiler Industry	Sensitivity to Detect Anomalies	Accuracy of Anomaly Detection	Data Used for Analysis	Possible Digital Markers	Associated Genomic Traits
Social Interactions	Monitoring group dynamics, identifying social stressors	High sensitivity in detecting changes in group dynamics that may indicate stress or health issues.	Accuracy depends on the specific methods and technologies used, but generally high for detecting major changes in social interactions.	Social interaction data from sensors and cameras, demographic data, veterinary health records.	Changes in group dynamics, abnormal social behaviors.	Group III Secreted Phospholipase A2 (sPLA2-III), Vasotocin and Mesotocin Receptors, (HTR2C) and (DRD4)
Behavioral Patterns	Real-time tracking and analysis of individual and group behaviors.	High sensitivity in detecting changes in behavior patterns that may indicate stress or health issues.	Accuracy depends on the specific methods and technologies used, but generally high for detecting major changes in behavior.	Behavioral data from sensors and cameras, accelerometer training data, demographic data, veterinary health records.	Changes in activity levels, abnormal behaviors.	MC4R gene, insulin signaling pathways, thyroid hormone pathways, and growth hormone pathways, serotonergic activity, genes related to hypothalamic-pituitary-adrenal (HPA) axis
Health-Related Phenotypes	Continuous monitoring of physical health parameters, developing disease phenotypes.	High sensitivity in detecting changes in health parameters that may indicate disease.	Accuracy depends on the specific methods and technologies used, but generally high for detecting major health issues.	Health data from sensors and cameras, demographic data, veterinary health records.	Changes in physical health parameters, signs of disease.	Growth hormone (GH), insulin-like growth factor (IGF), AMPD1 gene, involved in energy metabolism, Major Histocompatibility Complex (MHC), Myostatin (MSTN), insulin-like growth factor 2 (IGF2), and growth hormone receptor (GHR), TLR (Toll-like receptor) genes, chicken Mx gene, an antiviral gene.
Resilience	Tracking responses to environmental stressors, predicting resilience.	Moderate sensitivity in detecting changes in resilience based on responses to environmental stressors.	Accuracy depends on the specific methods and technologies used, but generally moderate for predicting resilience.	Behavioral data from sensors and cameras, environmental data, demographic data, veterinary health records.	Changes in response to environmental stressors, signs of stress.	Hypothalamic-Pituitary-Adrenal (HPA) Axis, Genetic variation in HSP genes, HSP70, serotonin receptor gene HTR2C and the dopamine receptor gene DRD4, mt-COI gene, a mitochondrial gene, cytokine genes
Affective States	Defining measurement paradigms for affective states, identifying symptoms of diverse physiological conditions.	High sensitivity in detecting changes in affective states that may indicate stress or health issues.	Accuracy depends on the specific methods and technologies used, but generally high for detecting major changes in affective states.	Behavioral data from sensors and cameras, physiological data, demographic data, veterinary health records.	Changes in affective states, signs of stress or discomfort.	Brain-Derived Neurotrophic Factor (BDNF) gene, corticotrophin-releasing hormone (CRH), serotonin transporter gene (SERT) and dopamine receptor genes (e.g., DRD2, DRD4), Major Histocompatibility Complex (MHC) or Interleukins genes, Genes involved in mesotocin and vasotocin pathways.

**Table 2:** Comprehensive Breakdown of Digital Phenotyping Platform Components for Enhanced Broiler Health Monitoring: Insights, Benefits, and Solutions.

Component	Description	Benefits	Challenges	Potential Solutions
Computational Pipelines	High-throughput pipelines analyze sensor and camera data to quantify broiler phenotypes.	Decodes complex data sets for informed decision making.	Requires shared expertise for design.	Collaborative design process considering industry’s priority measurement needs.
Modular Design Principles	The pipeline creation process is formulaic and modular for efficiency and standardization.	Swift and effective creation of new pipelines for broader adoption.	-	Deployment of existing tools on research cyberinfrastructure.
Machine Learning Integration	Machine learning techniques analyze complex data sets and draw insights.	Automates and enhances accuracy of phenotype analysis.	-	Use of supervised and unsupervised learning algorithms.
Research Cyberinfrastructure Integration	Leverages cloud computing resources, distributed computing frameworks, and data storage solutions.	Facilitates scalability and accessibility, reducing computational time.	-	Utilization of cloud computing resources and distributed computing frameworks.
Real-time Health Monitoring	The platform allows for real-time monitoring of broiler health.	Boosts productivity and contributes towards improved animal welfare.	Requires reliable and continuous data collection.	Use of reliable sensor technology and advanced camera systems.
Data-Driven Decision Making	The platform aids in optimizing management strategies.	Enhances overall broiler performance and monitors environmental impacts.	Farmers may need training to understand and use the data.	Provide training and support to farmers.
Animal Welfare Improvement	The platform enables early detection of health disorders and optimizing management strategies.	Leads to increased productivity and meets demand for ethically sourced products.	Balancing improved welfare with cost-effective production.	Optimization of management strategies and productivity improvement.
Enhanced Productivity	The platform enables the selection of healthier and more productive broiler lines.	Leads to improved genetic traits and overall flock performance.	Requires reliable and accurate phenotype analysis.	Use of efficient computational pipelines and machine learning techniques.
